# Correction: Integration of wearable devices and artificial intelligence in Alzheimer’s disease: A scoping review protocol

**DOI:** 10.1371/journal.pone.0339031

**Published:** 2025-12-12

**Authors:** The PLOS One Staff

The tenth author, Qian Xu, is incorrectly noted as the corresponding author. The correct corresponding author is Shihua Cao. Shihua Cao’s email address is: csh@hznu.edu.cn.

The publisher apologizes for the error.
