# Supplementary material for: Integration of wearable devices and artificial intelligence in Alzheimer’s disease: A scoping review protocol
Source: PLoS One. 2025 Sep 12;20(9):e0331129. doi: 10.1371/journal.pone.0331129 (PMC12431128; doi:10.1371/journal.pone.0331129)
Supplement: S2 Table — (DOCX) [file pone.0331129.s002.docx]

**Supplementary Table 2. Search strategies for different databases.**

| **Database** | **Search strategies for different databases** |
| --- | --- |
| **Web of Science Core Collection** | (TS=("Cognitive Impairment" OR "Mild Cognitive Impairment" OR "Alzheimer's Disease" OR "Dementia" ) AND TS=("Digital Health Technology" OR "Digital Health" OR "gait" OR "smart watch" OR "Band" OR "hand movement*" OR "manual movement*" OR "eye movement*" OR "digital measure" OR "Sleep Monitoring" OR "Naturalistic driving" OR "Global positioning system" OR "wearable device" OR "wearable*" OR "Driving behavior" OR "Sensor" OR " Inertia Measurement Unit" OR "eye tracker" or "eye-tracking device" OR "mobile EEG" OR "wireless EEG" OR "portable EEG" OR "wearable EEG" OR "VR" OR "Virtual Reality" OR "Heart Rate Variability" OR "heart rate")) AND (TS=( "diagnosis" OR "evaluation" OR "assessment" OR "prediction" OR "forecasting" OR "Artificial intelligence" OR"machine learning" OR "deep learning" OR"forecasting model*" OR "prediction model*")) |
| **Pubmed** | ((Cognitive Impairment[MeSH Terms]) OR (Mild Cognitive Impairment) OR (Alzheimer's Disease[MeSH Terms]) OR (Dementia[MeSH Terms])) AND ((Digital Health Technology) OR (Digital Health[MeSH Terms]) OR (gait[MeSH Terms]) OR (smart watch) OR (Band) OR (hand movement*) OR (manual movement*) OR (eye movement[MeSH Terms]) OR (digital measure) OR (Sleep Monitoring) OR (Naturalistic driving) OR (Global positioning system[MeSH Terms]) OR (wearable device[MeSH Terms]) OR (Driving behavior) OR (Sensor) OR (Inertia Measurement Unit) OR (mobile EEG) OR (wireless EEG) OR (portable EEG) OR (wearable EEG)) AND ( (diagnosis[MeSH Terms]) OR (evaluation) OR (assessment) OR (prediction) OR (forecasting[MeSH Terms]) OR(Artificial intelligence[MeSH Terms]) OR (machine learning[MeSH Terms]) OR (deep learning[MeSH Terms]) OR (forecasting model*) OR (prediction model*) ) |
| **Embase** | ('Artificial intelligence':ti,ab,kw OR 'machine learning':ti,ab,kw OR 'deep learning':ti,ab,kw OR 'prediction':ti,ab,kw OR 'forecasting':ti,ab,kw OR 'diagnosis':ti,ab,kw OR 'evaluation':ti,ab,kw OR 'assessment':ti,ab,kw) AND ( 'gait':ti,ab,kw OR 'smart watch':ti,ab,kw OR 'band':ti,ab,kw OR 'hand movement':ti,ab,kw OR 'manual movement':ti,ab,kw OR 'eye movement':ti,ab,kw OR 'digital measure':ti,ab,kw OR 'sleep monitoring':ti,ab,kw OR 'global positioning system':ti,ab,kw OR 'wearable device':ti,ab,kw OR 'driving behavior':ti,ab,kw OR 'sensor':ti,ab,kw OR 'inertia measurement unit':ti,ab,kw OR 'eye tracker':ti,ab,kw OR 'eye-tracking device':ti,ab,kw OR 'mobile eeg':ti,ab,kw OR 'wireless eeg':ti,ab,kw OR 'portable eeg':ti,ab,kw OR 'wearable eeg':ti,ab,kw) AND('mild cognitive impairment':ti,ab,kw OR 'alzheimers disease':ti,ab,kw OR 'dementia':ti,ab,kw OR 'cognitive impairment':ti,ab,kw OR 'forecasting model':ti,ab,kw OR 'prediction model':ti,ab,kw ) |
| **IEEE Explore** | [((All Metadata:"Cognitive Impairment" ) OR (All Metadata:"Mild Cognitive Impairment") OR (All Metadata:"Alzheimer's Disease") OR (All Metadata:"Dementia" ))) AND ((All Metadata:"Digital Health Technology") OR (All Metadata:"wearable device") OR (All Metadata:"gait" ) OR (All Metadata:"smart watch") OR (All Metadata:"Inertia Measurement Unit") OR (](https://ieeexplore.ieee.org/search/searchresult.jsp?contentType=all&refinements=ContentType:Journals&sortType=&filter=-ContentType+EQ+"Newsletters"&searchField=Search_All&combineQuery=sortType=&filter=-ContentType+EQ+"Newsletters"&searchField=Search_All.OPAND.filter=-ContentType+EQ+"Newsletters"&matchBoolean=true&searchField=Search_All&queryText=(Search_All:"Cognitive+Impairment"+)+OR+(Search_All:"Mild+Cognitive+Impairment")+OR+(Search_All:"Alzheimer's+Disease")+OR+(Search_All:Dementia+).OPAND.filter=-ContentType+EQ+"Newsletters"&matchBoolean=true&searchField=Search_All&queryText=(Search_All:"digital+biomarker")+OR+(Search_All:"electronic+biomarker")+OR+(Search_All:"e-biomarker")+OR+(Search_All:"wearable+biomarker")+OR+(Search_All:"mobile+biomarker")+OR+(Search_All:"sensor-based+biomarker")+OR+(Search_All:gait+)+OR+(Search_All:smartphone)+OR+(Search_All:computer)+OR+(Search_All:tablet)+OR+(Search_All:"smart+watch").OPAND.filter=-ContentType+EQ+"Newsletters"&matchBoolean=true&searchField=Search_All&queryText=(Search_All:detection)+OR+(Search_All:screening)+OR+(Search_All:"screening+test*")+OR+(Search_All:"computerised+screening+test*")+OR+(Search_All:diagnosis)+OR+(Search_All:"evaluation")+OR+(Search_All:"assessment")&history=no)[All Metadata:"mobile EEG") OR (All Metadata:"wireless EEG") OR (All Metadata:"portable EEG") OR (All Metadata:"wearable EEG")](https://ieeexplore.ieee.org/search/searchresult.jsp?contentType=all&refinements=ContentType:Journals&sortType=&filter=-ContentType+EQ+"Newsletters"&searchField=Search_All&combineQuery=filter=-ContentType+EQ+"Newsletters"&matchBoolean=true&searchField=Search_All&queryText=(Search_All:mobile+EEG)+OR+(Search_All:wireless+EEG)+OR+(Search_All:portable+EEG)+OR+(Search_All:wearable+EEG).OPAND.filter=-ContentType+EQ+"Newsletters"&matchBoolean=true&searchField=Search_All&queryText=(Search_All:"Cognitive+Impairment")+OR+(Search_All:"Mild+Cognitive+Impairment")+OR+(Search_All:"Alzheimer's+Disease")+OR+(Search_All:Dementia).OPAND.filter=-ContentType+EQ+"Newsletters"&matchBoolean=true&searchField=Search_All&queryText=(Search_All:detection)+OR+(Search_All:screening)+OR+(Search_All:"screening+test*")+OR+(Search_All:"computerised+screening+test*")+OR+(Search_All:diagnosis)+OR+(Search_All:"evaluation")+OR+(Search_All:"assessment")&history=no)[)) AND ((All Metadata:"detection") OR (All Metadata:"screening") OR (All Metadata:"forecasting") OR (All Metadata:"prediction model") OR (All Metadata:"diagnosis") OR (All Metadata:"evaluation") OR (All Metadata:"assessment") OR (All Metadata:"prediction") OR (All Metadata:"Artificial intelligence") OR (All Metadata:"machine learning") OR (All Metadata:"deep learning")](https://ieeexplore.ieee.org/search/searchresult.jsp?contentType=all&refinements=ContentType:Journals&sortType=&filter=-ContentType+EQ+"Newsletters"&searchField=Search_All&combineQuery=sortType=&filter=-ContentType+EQ+"Newsletters"&searchField=Search_All.OPAND.filter=-ContentType+EQ+"Newsletters"&matchBoolean=true&searchField=Search_All&queryText=(Search_All:"Cognitive+Impairment"+)+OR+(Search_All:"Mild+Cognitive+Impairment")+OR+(Search_All:"Alzheimer's+Disease")+OR+(Search_All:Dementia+).OPAND.filter=-ContentType+EQ+"Newsletters"&matchBoolean=true&searchField=Search_All&queryText=(Search_All:"digital+biomarker")+OR+(Search_All:"electronic+biomarker")+OR+(Search_All:"e-biomarker")+OR+(Search_All:"wearable+biomarker")+OR+(Search_All:"mobile+biomarker")+OR+(Search_All:"sensor-based+biomarker")+OR+(Search_All:gait+)+OR+(Search_All:smartphone)+OR+(Search_All:computer)+OR+(Search_All:tablet)+OR+(Search_All:"smart+watch").OPAND.filter=-ContentType+EQ+"Newsletters"&matchBoolean=true&searchField=Search_All&queryText=(Search_All:detection)+OR+(Search_All:screening)+OR+(Search_All:"screening+test*")+OR+(Search_All:"computerised+screening+test*")+OR+(Search_All:diagnosis)+OR+(Search_All:"evaluation")+OR+(Search_All:"assessment")&history=no)OR (All Metadata:"forecasting model")OR (All Metadata:"prediction model"))  You Refined By: Content Type: Journals |
| **CINAHL** | ( "gait" OR "eye movement*" OR "digital measure" OR "Naturalistic driving" OR "Global positioning system" OR "wearable device*" OR "smart watch" OR "Sleep Monitoring" OR "Driving behavior" OR Sensor OR "mobile applications" ) OR ( "eye tracker" OR "eye-tracking device" OR "mobile EEG" OR "wireless EEG" OR "portable EEG" OR "wearable EEG" ) )AND ("Cognitive Impairment" OR "Mild Cognitive Impairment" OR "Alzheimer's Disease" OR "Dementia") AND("forecasting" OR "diagnosis" OR "evaluation" OR "assessment" OR "prediction" OR "Artificial intelligence" OR"machine learning" OR "deep learning" OR "forecasting model" OR "prediction model") |
