# Supplementary material for: Integration of wearable devices and artificial intelligence in Alzheimer’s disease: A scoping review protocol
Source: PLoS One. 2025 Sep 12;20(9):e0331129. doi: 10.1371/journal.pone.0331129 (PMC12431128; doi:10.1371/journal.pone.0331129)
Supplement: S4 Table — (DOCX) [file pone.0331129.s004.docx]

**Supplementary Table 4.** **Charting data form**

| **Charting Dimensions** | **Aspects** |
| --- | --- |
| **General study information** |  |
| General information | Year of publication, Aim of study |
| **Methodological characteristics of included studies** |  |
| Data source | Data source category: systematic reviews, clinical trial, prospective cohort, retrospective cohort, cross-sectional study, databases registries, medical records. Description of the data source. Period |
| Study population | Participants description, Total study sample size |
| Location | Country and regions |
| Prediction modelling | Number of models used |
| Model development | Specific type of AI model used (e.g., neural network, SVM, decision tree, etc.). |
| Period of prediction | For example, years, days |
| Type of prediction modelling studies | Prediction model development without external validation in independent data, Prediction model development with external validation in independent data, External model validation, possibly with model updating |
| Number and type of predictors | Variables evaluated for their association with the outcome of interest (e.g., demographics and disease characteristics) |
| Multimodal fusion strategies | Types of features and specific fusion techniques, such as early fusion and late fusion. |
| Predicted outcome | New cases; under-reporting cases; new confirmed cases, Study description: description of the cases that will be assessed |
| Missing data | Number of participants with missing data for each predictor, Handling of missing data (e.g., complete case analysis, imputation, or other methods) |
| Was under-reporting considered? | Yes, no or unclear, How was it considered in the model (predictor or outcome) |
| Statistical approaches | Types of statistical approaches |
| Model predictive performance | Calibration (calibration plot, calibration slope and Hosmer-Lemeshow test) and discrimination (C-statistic, D-statistic and log-rank) measures with CIs, if applicable |
| Type of validation | What type of validation the authors used? (e.g., train test split,k-fold cross validation, external validation) |
| Evaluation metrics | Metrics used to evaluate the model's performance (e.g., accuracy, precision, etc.). |
| Formats of presenting models | Formats including tables, figures, formulas, and multiple formats |
| Software | Any software used to build the model |
| **Reproducibility and Reporting Standards** |  |
| Code Availability | Whether the study provided open-source code or not. |
| Reporting Standards Used | The specific reporting guidelines or standards adhered to by the study, such as TRIPOD |
| Conclusion | Main conclusion |
