# Supplementary material for: Integration of wearable devices and artificial intelligence in Alzheimer’s disease: A scoping review protocol
Source: PLoS One. 2025 Sep 12;20(9):e0331129. doi: 10.1371/journal.pone.0331129 (PMC12431128; doi:10.1371/journal.pone.0331129)
Supplement: S5 Table — (DOCX) [file pone.0331129.s005.docx]

**Supplementary Table 5. PROBAST Form.**

| **DOMAIN 1: Participants** | | | |
| --- | --- | --- | --- |
| **A. Risk of Bias** | | | |
| *Describe the sources of data and criteria for participantselection:* | | | |
|  | | Dev | Val |
| 1.1 Were appropriate data sources used, e.g. cohort, RCT or nested case-control study data? | |  |  |
| 1.2 Were all inclusions and exclusions of participants appropriate? | |  |  |
| **Risk of bias introduced by selection of participants** | **RISK:**  *(low/ high/ unclear)* |  |  |
| *Rationale of bias rating:* | | | |
|  | | | |
| **B. Applicability** | | | |
| *Describe included participants, setting and dates:* | | | |
| **Concern that the included participants and setting do not match the review question** | **CONCERN:**  *(low/ high/ unclear)* |  |  |
| *Rationale of applicability rating:* | | | |
|  | | | |

| **DOMAIN 2: Predictors** | | | |
| --- | --- | --- | --- |
| **A. Risk of Bias** | | | |
| *List and describe predictors included in the final model, e.g. definition and timing of assessment:* | | | |
|  | | Dev | Val |
| 2.1 Were predictors defined and assessed in a similar way for all participants? | |  |  |
| 2.2 Were predictor assessments made without knowledge of outcome data? | |  |  |
| 2.3 Are all predictors available at the time the model is intended to be used? | |  |  |
| **Risk of bias introduced by predictors or their assessment** | **RISK:**  *(low/ high/ unclear)* |  |  |
| *Rationale of bias rating:* | | | |
| **B. Applicability** | | | |
| Concern that the definition, assessment or timing of predictors in the model do not match the review question | **CONCERN:**  *(low/ high/ unclear)* |  |  |
| *Rationale of applicability rating:* | | | |

| **DOMAIN 3: Outcome** | | | |
| --- | --- | --- | --- |
| **A. Risk of Bias** | | | |
| *Describe the outcome, how it was defined and determined, and the time interval between predictor assessment and outcome determination:* | | | |
|  | | Dev | Val |
| 3.1 Was the outcome determined appropriately? | |  |  |
| 3.2 Was a pre-specified or standard outcome definition used? | |  |  |
| 3.3 Were predictors excluded from the outcome definition? | |  |  |
| 3.4 Was the outcome defined and determined in a similar way for all participants? | |  |  |
| 3.5 Was the outcome determined without knowledge of predictor information? | |  |  |
| 3.6 Was the time interval between predictor assessment and outcome determination appropriate? | |  |  |
| **Risk of bias introduced by the outcome or its determination** | **RISK:**  *(low/ high/ unclear)* |  |  |
| *Rationale of bias rating:* | | | |
| **B. Applicability** | | | |
| *At what time point was the outcome determined:*  *If a composite outcome was used, describe the relative frequency/distribution of each contributing outcome:* | | | |
| **Concern that the outcome, its definition, timing or determination do not match the review question** | **CONCERN:**  *(low/ high/ unclear)* |  |  |
| *Rationale of applicability rating:* | | | |
