# Supplementary material for: Integration of wearable devices and artificial intelligence in Alzheimer’s disease: A scoping review protocol
Source: PLoS One. 2025 Sep 12;20(9):e0331129. doi: 10.1371/journal.pone.0331129 (PMC12431128; doi:10.1371/journal.pone.0331129)
Supplement: Attachment — Submitted filename: Response to the reviewers comments.docx [file pone.0331129.s007.docx]

March 30, 2025

Dear Editor,
Dear Reviewers,

Dear PLOS One Editorial Office,

We have submitted the revised manuscript entitled "Integration of wearable devices and artificial intelligence in Alzheimer's disease: a scoping review protocol" (Submission ID: PONE-D-25-03879R1). Additionally, we have provided a version of the manuscript with tracked changes for review.

We sincerely appreciate the efforts of the editorial team and the constructive feedback provided by the reviewers. The suggestions were extremely valuable and played a crucial role in improving the quality of our manuscript. These recommendations have been essential in guiding our research. We have made revisions in response to each comment from the four reviewers, and we have reorganized the content in the attached document accordingly.

1.Given the increasing popularity of integrating AI with wearable devices, it has become more important than ever to write a review on Alzheimer’s disease in this context.

2.To our knowledge, this manuscript represents the first scoping review specifically focusing on AI models for wearable devices in Alzheimer’s disease.

3.We anticipate that executing this protocol will yield meaningful insights that will contribute to clinical applications, such as model validation and performance distribution.

We hope that this revised manuscript meets the expectations of both the journal and the reviewers, ultimately allowing it to reach a broader audience. Our goal is to facilitate knowledge transfer in this field and make a significant contribution to its development. Therefore, we are hopeful that the revised manuscript will be accepted for publication in PLOS One.

Corresponding Author: Shihua Cao

Email: [csh@hznu.edu.cn](mailto:*csh@hznu.edu.cn)

Key Laboratory of Cognitive Disorder Assessment Technology

Hangzhou, China

-------------------------------------------------------------------------------------------------------

1. When submitting your revision, we need you to address these additional requirements.

Please ensure that your manuscript meets PLOS ONE's style requirements, including those for file naming. The PLOS ONE style templates can be found athttps://journals.plos.org/plosone/s/file?id=wjVg/PLOSOne_formatting_sample_main_body.pdf and<https://journals.plos.org/plosone/s/file?id=ba62/PLOSOne_formatting_sample_title_authors_affiliations.pdf>

**Response to Editor's comment 1:**

We appreciate the editor's comments regarding the manuscript formatting. We have made the necessary adjustments in accordance with the journal's formatting guidelines. Specifically, we have:Added table titles following the references section. Modified the font sizes for section, subsection, and sub-subsection headings. Bolded the prefix of the figure titles. Ensured that all headings are written in sentence case, as per the journal’s requirements.

-----------------------------------------------------------------------------------------------------

2. Thank you for stating the following financial disclosure: [Zhejiang Province Traditional Chinese Medicine, Science, and Technology Project (2023ZF134) supported by SC, Zhejiang Provincial Medical and Health Science and Technology Program Project (2022KY1052) supported by SC,First-Class Course of Zhejiang Province (2022-1133) supported by SC,Basic Public Welfare Research Project/Joint Fund Project of Zhejiang Province: Research on accurate localization of gait disorder brain region in Parkinson's disease based on pfMRI data set and hierarchical Bayesian model(LBY23H200002), supported by XL.].

Please state what role the funders took in the study.  If the funders had no role, please state: ""The funders had no role in study design, data collection and analysis, decision to publish, or preparation of the manuscript.""If this statement is not correct you must amend it as needed.Please include this amended Role of Funder statement in your cover letter; we will change the online submission form on your behalf.

**Response to Editor's comment 2:**

We appreciate the editor's suggestion regarding the funding statement. In response, we have added the following statement in the Acknowledgments section: "The funders had no role in study design, data collection and analysis, decision to publish, or preparation of the manuscript." We also included a statement about the role of funders in the second-to-last paragraph of the cover letter: "The funders had no role in study design, data collection and analysis, decision to publish, or preparation of the manuscript."

----------------------------------------------------------------------------------------------------

3. When completing the data availability statement of the submission form, you indicated that you will make your data available on acceptance. We strongly recommend all authors decide on a data sharing plan before acceptance, as the process can be lengthy and hold up publication timelines. Please note that, though access restrictions are acceptable now, your entire data will need to be made freely accessible if your manuscript is accepted for publication. This policy applies to all data except where public deposition would breach compliance with the protocol approved by your research ethics board. If you are unable to adhere to our open data policy, please kindly revise your statement to explain your reasoning and we will seek the editor's input on an exemption. Please be assured that, once you have provided your new statement, the assessment of your exemption will not hold up the peer review process.

**Response to Editor's comment 3:**

We appreciate the editor's reminder regarding the data statement. All authors have agreed to the statement that all data in the manuscript will be made freely accessible.

-------------------------------------------------------------------------------------------------------

4. PLOS requires an ORCID iD for the corresponding author in Editorial Manager on papers submitted after December 6th, 2016. Please ensure that you have an ORCID iD and that it is validated in Editorial Manager. To do this, go to ‘Update my Information’ (in the upper left-hand corner of the main menu), and click on the Fetch/Validate link next to the ORCID field. This will take you to the ORCID site and allow you to create a new iD or authenticate a pre-existing iD in Editorial Manager.

**Response to Editor's comment 4:**

We appreciate the editor's reminder. The corresponding author has now verified and linked their ORCID. Finally, we would like to express our sincere gratitude to the editor for the meticulous review, which has helped us refine the formatting details of the manuscript and assisted us in adhering to the journal's required declarations.

------------------------------------------------------------------------------------------------------

Reviewer #1: My concerns are as follows:

1. The abstract is overly redundant and appears to be written as an introduction; a complete rewrite of the abstract is recommended.

**Response to Reviewer #1's comment 1:**

We appreciate your detailed comments on the abstract. Indeed, our original abstract was too lengthy, and as per your suggestion, we have rewritten it. This revision has significantly improved the clarity of the abstract. Thank you once again for your constructive feedback.

**Modified content:** The incidence of Alzheimer's disease continues to rise, and predictive models combining artificial intelligence (AI) with wearable devices offer a new approach for its detection and diagnosis. Given the rapid development of these emerging technologies, a comprehensive review is necessary to supplement the evidence in this field. This review aims to systematically evaluate AI-based predictive models using wearable devices, with a focus on their measurement outcomes and model development processes. Following the Arksey and O'Malley framework and incorporating PRISMA and its extension guidelines, this study will search multiple databases, including Web of Science, Cochrane Library, and PubMed, covering relevant gray literature. The quality of the included studies will be rigorously assessed using the Prediction Model Risk of Bias Assessment Tool (PROBAST) and Transparent Reporting of a Multivariable Prediction Model for Individual Prognosis or Diagnosis (TRIPOD) checklists. Two independent reviewers will conduct title and abstract screening, retrieve and assess full-text evidence sources, and extract data. The results will be narratively synthesized and presented in tables and figures. This review is expected to provide systematic evidence to support predictive models combining wearable devices and AI, contributing to the advancement of this field.

**(Lines 26-42)**

------------------------------------------------------------------------------------------------------
2. The paper explores a relatively emerging intersection of wearable devices and AI in Alzheimer's disease (AD). However, the existing literature on AI in healthcare, including Alzheimer's, has been growing, and the manuscript does not highlight its unique contribution clearly in the introduction. It would benefit from a clearer statement on how the review will fill existing gaps.

**Response to Reviewer #1's comment 2:**

We greatly appreciate your detailed and constructive feedback on our research. The previous version of the manuscript indeed lacked sufficient emphasis on how our work addresses existing research gaps. In response to your suggestion, we have revised the introduction to highlight the contributions of our study and how it fills these gaps. This addition effectively clarifies our research's unique contribution to the field.

(1)First, we have outlined the limitations of existing studies and explained how their focus differs from ours.

**Modified content:** The potential of these technologies is being explored; however, only two systematic reviews [19, 20] have addressed the application of wearable devices and AI models in AD. These reviews cover a broader range of topics and primarily focus on studies related to clinical biomarker data, such as radiomics. They do not provide detailed reports on the deployment strategies of wearable devices or the specifics of model construction. Additionally, these reviews fail to adequately demonstrate the feasibility of integrating wearable devices with AI technologies.

**(Lines 96-102)**

(2)We have also added a section outlining the contributions of our study and the gaps it addresses.

**Modified content:** The unique contribution of this protocol lies in its development of a systematic evaluation and comprehensive review of AI prediction models based on wearable devices. By providing a comprehensive review of existing research, it addresses the gap in the current literature concerning the integration of these two technologies. Specifically, we aim to report on several key dimensions, including the selection of wearable devices, data collection paradigms, feature selection strategies, model construction and training, model validation, and model transparency. In addition to analyzing the applications of various wearable devices, we explore how advanced AI techniques can enhance their diagnostic accuracy and monitoring capabilities. This approach offers new perspectives for the early diagnosis of AD, prediction of disease progression, and personalized healthcare.

**(Lines 111-121)**

------------------------------------------------------------------------------------------------------

3. The protocol promises to synthesize a significant amount of existing research, but it remains unclear what specific contributions it will make beyond summarizing previous findings. It would be useful to further emphasize how the findings will directly influence future research directions or clinical practices. Some workss are worth mentioning: (1)DOI:10.62762/CJIF.2024.876830 (2)DOI:10.62762/TIS.2024.680959

**Response to Reviewer #1's comment 3:**

(1) We greatly appreciate your suggestion regarding the specific contributions of our manuscript. Due to our previous unclear statement, this section was insufficient. In response to your feedback, we have made the necessary revisions. We have expanded the discussion section to include the contributions of the content we have collected to clinical practice and future research. Thank you once again for this specific and valuable suggestion, which has greatly improved the quality of our manuscript.

**Modified content:** Specifically, we anticipate providing a series of clinically meaningful insights. First, the summary of wearable devices and data collection paradigms will highlight the practical applications and limitations of current technologies in AD diagnosis. For instance, the pairing of different types of devices with various tasks will guide the design of clinical diversity, while the identification of causes for data gaps during the collection process will help recognize potential issues with existing data acquisition technologies. Furthermore, these insights can provide direction for future improvements in device design and data collection methods. The analysis of the quantity, types, and performance distributions of the algorithms currently in use will provide a clear representation of the field's status and development trends. By examining the application of different algorithm models and their performance across various datasets, we can identify which algorithms perform best in AD diagnosis and prediction tasks, as well as which models still face performance bottlenecks. This study will also reveal the applicability of various models in different clinical scenarios (e.g., model training costs, device portability, and data acquisition challenges), helping to identify which models are best suited for early diagnosis and disease progression tracking. Although we do not anticipate directly comparing the performance of unimodal and multimodal models, but rather presenting the performance distribution (due to the objective influences of differences in data acquisition devices, tasks, feature types, and algorithm architectures), this approach will still provide valuable insights into the combination of multimodal data. Furthermore, an analysis of external validation, calibration, and related aspects will help assess the generalizability and reliability of these models across different datasets and real-world clinical environments. The public release of code is essential for promoting transparency and reproducibility within the field. In summary, this scoping review will not only highlight the strengths of current technologies in the field of AD but will also assist researchers and clinicians in understanding the challenges that may arise in practical applications.

**(Lines 398-427)**

(2)The two studies you mentioned regarding EEG data processing are indeed highly relevant to the design of our protocol. In response, we have added these two studies in the introduction to enrich the theoretical background on the progress between machine learning algorithms and data processing. Your suggestion has greatly helped improve the completeness of our manuscript. Thank you once again for this constructive feedback.

**Modified content:** Similarly, graph neural networks play a crucial role in the spatiotemporal processing of EEG data by simultaneously considering the dynamic changes in the time series and the spatial relationships between different brain regions[17,18].

**(Lines 92-95)**

------------------------------------------------------------------------------------------------------

4. The manuscript discusses the types of data (e.g., gait, EEG) that will be covered, but there is limited discussion on how diverse data collection methods could influence the model's performance. It should address how the variety of data sources will be handled, especially multimodal data.

**Response to Reviewer #1's comment 4:**

We greatly appreciate your specific suggestions regarding the data processing of different models in our manuscript. While we had considered multimodal and different data collection, it was not sufficiently presented in the original manuscript. In response, we have added a more detailed description of the data processing in the Data Synthesis section. This revision strengthens our research methodology and enriches the content of our study. Thank you once again for your valuable suggestions.

**Modified content:** Data Sources: After extracting the data types for the study, the models are initially categorized into unimodal and multimodal models based on the nature of the modalities. For unimodal models, further classification is performed based on the specific data sources captured (e.g., variations in the type of wearable devices and their placement locations lead to differences in the types of data collected). These models are expected to include gait models, manual models, physiological signal models, speech models, and possibly eye-tracking models. For multimodal models that involve multiple data types or integrate omics data, scale information, and other content, we will further discuss the fusion methods of the root modalities (early fusion, mid-fusion, late fusion, and hybrid fusion). Additionally, we will present the performance distribution differences between unimodal and multimodal models.

**(Lines 356-366)**

------------------------------------------------------------------------------------------------------

5. What are the newly-added values?

**Response to Reviewer #1's comment 5:**

We greatly appreciate your suggestions regarding the research value of our manuscript.

1)Firstly, this study will be the first scoping review specifically focusing on AI models for wearable devices in Alzheimer's disease. It will comprehensively evaluate these models using the PROBAST and TRIPOD tools, providing a clear overview of the current state of the field and offering guidance for the design and reporting of future studies.

2)As per your suggestion, we have added the specific content and significance of this study in the introduction. This will provide a valuable reference for the future development and deployment of these models.

3)In the discussion section, we have included the contributions of our research to future clinical practice and further research, as you recommended.

**The specific added value of our manuscript includes the following points:**

1)We provide the optimal algorithms and associated performance for these models, allowing readers to visually compare the performance differences between them, thus offering insights for future model development.

2)We outline the data collection processes, the devices used, and the paradigms employed, providing valuable information for researchers planning similar studies.

3)We highlight the differences in data types across various models, offering a clear comparison of performance differences in wearable device models.

4)We present the detailed construction aspects of these models, including whether missing values were handled, whether external validation was performed, and whether calibration was applied. These insights will offer standardized recommendations for future model construction.

5)As far as we know, this agreement is the first comprehensive review focusing on AI models for wearable devices in Alzheimer's disease.

------------------------------------------------------------------------------------------------------

6. The manuscript lacks a detailed discussion on the theoretical models that underpin the use of AI in AD prediction. A more comprehensive review of existing theories or frameworks (such as machine learning principles and their adaptation for healthcare applications) would strengthen the theoretical grounding.

**Response to Reviewer #1's comment 6:**

(1) We greatly appreciate your observation regarding the absence of existing theoretical models in our manuscript. In response to your suggestion, we have added a detailed discussion on the theoretical models related to the application of AI in Alzheimer's disease prediction in the introduction. Additionally, we have included fundamental theories of machine learning and revised Figure 1 to highlight the AI model construction methods and theories based on wearable devices for Alzheimer's disease. Your input on theoretical models has significantly improved the overall quality of our manuscript. We sincerely thank you once again for your valuable suggestions.

**Modified content:** With the rapid advancement of AI technology, machine learning has become a critical tool in the healthcare field, particularly in disease prediction and diagnosis[9]. Machine learning is a technique that trains models using data, enabling computers to automatically improve their performance based on experience. Machine learning methods can be categorized into three main types, depending on the learning approach: supervised learning, unsupervised learning, and reinforcement learning[10]. In the prediction of AD, wearable devices can provide various types of data (such as physiological signals, gait, and activity levels), and when integrated with machine learning, they enable high-frequency monitoring and personalized diagnosis [10]. Clinical prediction models use multiple variables to estimate current diagnostic outcomes (diagnostic models) or predict future disease outcomes (prognostic models). Several studies have developed predictive models for AD based on wearable devices. For example, Younghoon Jeon used SHIMMER wearable devices to monitor gait data and combined algorithms such as random forests and AdaBoost, achieving an accuracy rate of 72% [11]. Ahsan Shahzad developed a support vector machine model, achieving an accuracy rate of 70% [12]. Kyeonggu Lee utilized wearable EEG signals to build a multilayer perceptron model, achieving an accuracy rate of 74% [13]. In our recent study, we integrated multiple wearable sensors within a three-stage federated learning framework to develop a home-based AD detection system [14]. Additionally, we constructed the EmoMarker system using wearable microphones and depth cameras, achieving a classification accuracy of 81.13% by leveraging motion and acoustic data [15]. In the framework where AI is integrated with wearable devices, the selection and training of models are key to successful application. Typically, choosing an appropriate machine learning algorithm (such as deep learning, random forests, or support vector machines) and training it based on the characteristics of patient data and clinical needs can optimize the model’s predictive performance. For example, when the model needs to handle complex time-series data (such as gait studies in neurodegenerative diseases), methods like recurrent neural networks and long short-term memory networks have demonstrated superior performance[16]. Similarly, graph neural networks play a crucial role in the spatiotemporal processing of EEG data by simultaneously considering the dynamic changes in the time series and the spatial relationships between different brain regions[17,18].

**(Lines 64-95)**

(2) Finally, after making these revisions, we noticed that the length of the introduction had increased. As a result, we decided to merge and rewrite the first and second paragraphs for better conciseness.

**Modified content:** As the global population continues to age, the incidence and severity of dementia are increasing, posing a significant challenge to global health[1]. According to the World Health Organization, over 55 million people are affected by dementia[2], with Alzheimer's disease (AD) accounting for 60% to 70% of cases[3]. AD is primarily characterized by cognitive impairment, memory loss, and a range of motor function deficits[4]. Due to its complex etiology and mechanisms, effective treatments capable of fully reversing the disease course remain unavailable, placing a substantial burden on both society and families[5]. Therefore, early diagnosis and prediction are crucial for optimizing care management strategies and delaying disease progression. Currently, clinical diagnosis of AD mainly relies on neuropsychological assessments and biomarker testing based on the ATN framework[6]. However, these methods are costly, invasive, and complex to administer, and are prone to biases from patient self-reports and external factors[7]. With advancements in technology, wearable devices have emerged as a promising tool for early diagnosis. These devices offer non-invasive, high-frequency monitoring capabilities and provide new insights for more convenient health management, potentially serving as a valuable complement to existing diagnostic methods[8].

**(Lines 47-63)**

**-----------------------------------------------------------------------------------------------------**

Reviewer #2: This is a very interesting manuscript. The use of wearable devices and the use of artificial intelligence as an intervention in the management and prevention of Alzheimer's disease is welcomed.

**Response to Reviewer #2's comment:**

We sincerely appreciate your recognition of our research. I am truly happy to receive such high praise from you. We will continue to put in our best efforts in the future stages of this research and strive to complete this work to perfection.

--------------------------------------------------------------------------------------------------

Reviewer #3: Although the protocol described in this manuscript itself is well designed, it does not provide any new findings. The authors should perform a scoping review based on their own protocol and publish the results.

**Response to Reviewer #3's comment:**

We greatly appreciate your recognition of the design of our manuscript. As this is a protocol, we cannot be certain about the specific new findings at this stage. However, we have further expanded on the potential contributions to clinical practice. Additionally, we will continue to refine this research in the subsequent phases of our work and aim to publish our findings as soon as possible.

**The addition of this agreement could potentially further contribute to the clinical field if implemented.** Specifically, we anticipate providing a series of clinically meaningful insights. First, the summary of wearable devices and data collection paradigms will highlight the practical applications and limitations of current technologies in AD diagnosis. For instance, the pairing of different types of devices with various tasks will guide the design of clinical diversity, while the identification of causes for data gaps during the collection process will help recognize potential issues with existing data acquisition technologies. Furthermore, these insights can provide direction for future improvements in device design and data collection methods.

The analysis of the quantity, types, and performance distributions of the algorithms currently in use will provide a clear representation of the field's status and development trends. By examining the application of different algorithm models and their performance across various datasets, we can identify which algorithms perform best in AD diagnosis and prediction tasks, as well as which models still face performance bottlenecks. This study will also reveal the applicability of various models in different clinical scenarios (e.g., model training costs, device portability, and data acquisition challenges), helping to identify which models are best suited for early diagnosis and disease progression tracking. Although we do not anticipate directly comparing the performance of unimodal and multimodal models, but rather presenting the performance distribution (due to the objective influences of differences in data acquisition devices, tasks, feature types, and algorithm architectures), this approach will still provide valuable insights into the combination of multimodal data.

Furthermore, an analysis of external validation, calibration, and related aspects will help assess the generalizability and reliability of these models across different datasets and real-world clinical environments. The public release of code is essential for promoting transparency and reproducibility within the field. In summary, this scoping review will not only highlight the strengths of current technologies in the field of AD but will also assist researchers and clinicians in understanding the challenges that may arise in practical applications.

------------------------------------------------------------------------------------------------------

Reviewer #4: First off, congrats on putting together this study protocol. Now, as I was reading through, a couple of things popped up that you might want to think about a bit more:
Methods
One thing to consider clarifying is regarding the "gray literature" you plan to retrieve. Could you give a few examples of what you consider gray literature in this context? This would make your search strategy even more transparent.
You mention using the PROBAST and TRIPOD tools. It might be helpful to briefly mention what each of these tools helps you assess (e.g., risk of bias for PROBAST, reporting completeness for TRIPOD) directly in the methods section rather than just citing them.
Regarding the databases mentioned in the methodology versus the supplementary material, it's important to note that the main "Search strategy" section explicitly lists Google Scholar, Scopus, and Cochrane Library (along with other databases) as sources that will be searched. However, in Supplementary file, which provides example search strategies for different databases, a specific search strategy for Google Scholar, Scopus, or Cochrane Library is not explicitly shown. For transparency and reproducibility, it would be beneficial for the authors to include example search strings for these databases as well in the supplementary material.
Overall, I must say that this is a very well-put-together study protocol, and I genuinely enjoyed reading it. Addressing these minor points will enhance the clarity, transparency, and rigor of your protocol. To be honest, I see significant potential for publication here if these enhancements are made. Your detailed methodology and focus on a crucial area of research make this a valuable contribution. I wish you the best of luck with your scoping review.

**Response to Reviewer #4's comment:**

We greatly appreciate your high recognition of our manuscript. We apologize for any confusion caused by unclear expressions in our design process. In response to your feedback, we have made the necessary revisions.

(1)First, due to our oversight, the phrasing of our inclusion criteria was not as intended. We have corrected this and removed the requirement that studies must be published in peer-reviewed journals. In fact, AI is a rapidly developing field, and some advanced research is often presented first at conferences. Therefore, we also revised our exclusion criteria to exclude non-research papers (such as reviews). With these changes, we have now included conference papers and certain preprints. Additionally, we have defined the sources for gray literature that we plan to search.

Following your suggestions, these revisions have addressed some minor issues and have enhanced the overall rigor of our manuscript.

**Modified content:**Inclusion Criteria:

The study must involve the use of wearable devices.

The study must be written in English.

No restrictions on the type of predictive model (including diagnostic and prognostic models).

Predictive models constructed using machine learning or deep learning algorithms.

Exclusion Criteria:

Studies for which the full text is unavailable or incomplete.

Duplicate publications.

Books, editorial materials, reviews, and other non-research type studies.

Studies not focused on AD, instead emphasizing other dementia subtypes or mild cognitive impairment (MCI).

Predictive models based solely on traditional biomarkers (e.g., imaging biomarkers, blood biomarkers, cerebrospinal fluid biomarkers).

**(Lines 289-303)**

**Modified content:**"Gray literature" refers to documents that have not been formally published or peer-reviewed. These materials are often difficult to retrieve through traditional academic databases but still contain valuable research findings or information. We will retrieve available research from conference papers and proceedings, preprints, and dissertations through the Opengrey database.

**(Lines 241-245)**

(2)Additionally, we appreciate your thorough review of the supplementary materials. In response to your request, we have included the search strategies for other databases.

**Modified content:**

| **Google Scholar** | ("Alzheimer's Disease" OR "Alzheimer") AND ("wearable devices" OR "wearable sensors" OR "wearable technology") AND ("artificial intelligence" OR "AI" OR "machine learning" OR "deep learning" OR "neural networks") |
| --- | --- |
| **PsycINFO** | Alzheimer's Disease AND wearable devices AND artificial intelligence |
| **Cochrane Library** | ("Alzheimer's disease" OR "Alzheimer disease" OR "dementia") AND ("wearable device" OR "wearable technology" OR "wearable sensor" OR "wearable sensor technology") AND ("artificial intelligence" OR "AI" OR "machine learning" OR "deep learning" OR "neural network" OR "predictive model") AND ("diagnosis" OR "monitoring" OR "prediction" OR "assessment") |
| **Scopus** | TITLE-ABS-KEY("Alzheimer's disease" OR "Alzheimer disease" OR dementia) AND TITLE-ABS-KEY("wearable device" OR "wearable technology" OR "wearable sensor" OR "wearable sensor technology") AND TITLE-ABS-KEY("artificial intelligence" OR "AI" OR "machine learning" OR "deep learning" OR "neural network" OR "predictive model") AND TITLE-ABS-KEY("diagnosis" OR "monitoring" OR "prediction" OR "assessment") |
| **EBSCO** | ("Alzheimer's disease" OR "Alzheimer disease" OR dementia) AND ("wearable device" OR "wearable technology" OR "wearable sensor" OR "wearable sensor technology") AND ("artificial intelligence" OR "AI" OR "machine learning" OR "deep learning" OR "neural network" OR "predictive model") AND ("diagnosis" OR "monitoring" OR "prediction" OR "assessment") |
| **Opengrey** | ("Alzheimer's Disease) AND ("Wearable devices" OR "Wearable sensors) AND ("Artificial Intelligence" OR "Machine Learning" OR "Deep Learning" OR "Predictive model" OR "Classification model") |

(3)Finally, we would like to thank you for your suggestion to provide more specific details regarding the research tools. In response, we have added further explanations about these tools. This revision has indeed improved the accuracy of our research and enhanced the quality of the methodology

**Modified content:**The PROBAST tool is used to assess the risk of bias in prediction models, helping to evaluate whether there is bias in the design, data processing, and analytical methods of the prediction models in research. The TRIPOD tool is used to assess and ensure the completeness of the reporting for multivariable prediction models. It provides a standardized reporting guideline that helps researchers clearly and transparently report the construction, validation process, and results of prediction models. The combined use of PROBAST and TRIPOD offers a more comprehensive framework to ensure the quality and transparency of predictive model research.

**(Lines 184-192)**
