# Supplementary material for: Integration of wearable devices and artificial intelligence in Alzheimer’s disease: A scoping review protocol
Source: PLoS One. 2025 Sep 12;20(9):e0331129. doi: 10.1371/journal.pone.0331129 (PMC12431128; doi:10.1371/journal.pone.0331129)
Supplement: Attachment — Submitted filename: Round 2.Response to the reviewers comments.docx [file pone.0331129.s008.docx]

June 13th, 2025

Dear Editor,
Dear Reviewers,

Dear PLOS One Editorial Office,

We have submitted the revised manuscript entitled "Integration of wearable devices and artificial intelligence in Alzheimer's disease: a scoping review protocol" (Submission ID: PONE-D-25-03879R1). Additionally, we have provided a version of the manuscript with tracked changes for review.

We sincerely appreciate the efforts of the editorial team and the constructive feedback provided by the reviewers. The suggestions were extremely valuable and played a crucial role in improving the quality of our manuscript. These recommendations have been essential in guiding our research. We have made revisions in response to each comment from the four reviewers, and we have reorganized the content in the attached document accordingly.

1.Given the increasing popularity of integrating AI with wearable devices, it has become more important than ever to write a review on Alzheimer’s disease in this context.

2.To our knowledge, this manuscript represents the first scoping review specifically focusing on AI models for wearable devices in Alzheimer’s disease.

3.We anticipate that executing this protocol will yield meaningful insights that will contribute to clinical applications, such as model validation and performance distribution.

We hope that this revised manuscript meets the expectations of both the journal and the reviewers, ultimately allowing it to reach a broader audience. Our goal is to facilitate knowledge transfer in this field and make a significant contribution to its development. Therefore, we are hopeful that the revised manuscript will be accepted for publication in PLOS One.

Corresponding Author: Shihua Cao

Email: [csh@hznu.edu.cn](mailto:*csh@hznu.edu.cn)

Key Laboratory of Cognitive Disorder Assessment Technology

Hangzhou, China

-------------------------------------------------------------------------------------------------------

------------------------------------------------------------------------------------------------------

Editor Comments

Please, carefully consider new comments and also improve responses to Reviewer 1.

Thank you for your thoughtful and constructive feedback. We have carefully reviewed the comments from Reviewer #1 once again. We highly value Reviewer #1's suggestions, which are insightful and have greatly contributed to the improvement of our manuscript. We believe that many of the concerns raised in the previous round of revisions have already been addressed. In this round of revisions, we have taken the following steps:

1.We meticulously reviewed all the comments from Reviewer #1.

2.We made comprehensive revisions to address all the issues raised in the first round of feedback from Reviewer #1.

3.We have added detailed responses and clarifications in this revision, so Reviewer #1 can fully understand the changes made to the manuscript.

4.We hope that these revisions and responses meet the expectations of both the editor and Reviewer #1.

Once again, we sincerely appreciate your careful work and the opportunity to further elaborate on our research protocol. We look forward to your feedback.

-------------------------------------------------------------------------------------------------------

Reviewer #1: My concerns are as follows:

1. The abstract is overly redundant and appears to be written as an introduction; a complete rewrite of the abstract is recommended.

**Response to Reviewer #1's comment 1:**

Thank you for your detailed and constructive feedback. We have made revisions to the abstract in the previous round, and in this round of revisions, we have further refined it. We hope that these adjustments will meet your expectations.

a. We have thoroughly reviewed numerous scoping review protocols published in PLOS One, paying close attention to the structure of their abstracts and the journal's recommendations for writing the introduction.
b. Typically, these abstracts begin with 1-2 sentences providing the background, followed by one sentence highlighting the research gap, then one sentence outlining the research aim. They also describe the framework the study will follow, the databases to be searched, the method of reporting results, and the expected contributions. For example, see protocols like DOI:10.1371/journal.pone.0307491, DOI:10.1371/journal.pone.029223, DOI:10.1371/journal.pone.0278606, DOI:10.1371/journal.pone.0287872, DOI:10.1371/journal.pone.0273737, and DOI:10.1371/journal.pone.0314591.
c. Therefore, our abstract is highly aligned with those of scoping review protocols published in PLOS One, and the structure and logic of the abstract adhere to the journal's guidelines.
d. However, upon further reflection, we believe there is still room for improvement in our abstract. We have revised it once again, emphasizing the research gap more clearly, explicitly stating that the expanded version of the PRISMA guidelines is PRISMA-ScR, and reiterating that this study is a protocol.

**e.** We have restructured the abstract as per the points mentioned above. Should you have any further questions or suggestions, please let us know, and we will be happy to make additional modifications.

| **Research Background** | The incidence of Alzheimer's disease (AD) continues to rise, and predictive models combining artificial intelligence (AI) with wearable devices offer a new approach for its detection and diagnosis. |
| --- | --- |
| **Research Gap** | Existing reviews remain focused on traditional biomarkers, making it necessary to supplement the evidence in this field, particularly given the rapid advancements in wearable and AI technologies. |
| **Research Aim** | The scoping review protocol aims to systematically evaluate AI-based predictive models using wearable devices for AD, with a focus on their measurement outcomes and model development processes. |
| **Guidelines Followed** | The review will follow the Arksey and O'Malley framework and incorporate PRISMA-ScR guidelines. |
| **Data Retrieval Methods** | This study will search multiple databases, including Web of Science, Cochrane Library, and PubMed, covering relevant gray literature. |
| **Content Evaluation Methods** | The quality of the included studies will be rigorously assessed using the Prediction Model Risk of Bias Assessment Tool (PROBAST) and Transparent Reporting of a Multivariable Prediction Model for Individual Prognosis or Diagnosis (TRIPOD) checklists. |
| **Data Organization and Results Presentation** | Two independent reviewers will conduct title and abstract screening, retrieve and assess full-text evidence sources, and extract data. The results will be narratively synthesized and presented in tables and figures. |
| **Expected Contributions of the Review** | The knowledge gained from this review is expected to provide systematic evidence supporting AI-based predictive models that combine wearable devices for AD, potentially offering insights into model construction details such as data collection and external validation. |

**f.Modified content:**The incidence of Alzheimer's disease (AD) continues to rise, and predictive models combining artificial intelligence (AI) with wearable devices offer a new approach for its detection and diagnosis. Existing reviews remain focused on traditional biomarkers, making it necessary to supplement the evidence in this field, particularly given the rapid advancements in wearable and AI technologies. The scoping review protocol aims to systematically evaluate AI-based predictive models using wearable devices for AD, with a focus on their measurement outcomes and model development processes. The review will follow the Arksey and O'Malley framework and incorporate PRISMA-ScR guidelines. This study will search multiple databases, including Web of Science, Cochrane Library, and PubMed, covering relevant gray literature. The quality of the included studies will be rigorously assessed using the Prediction Model Risk of Bias Assessment Tool (PROBAST) and Transparent Reporting of a Multivariable Prediction Model for Individual Prognosis or Diagnosis (TRIPOD) checklists. Two independent reviewers will conduct title and abstract screening, retrieve and assess full-text evidence sources, and extract data. The results will be narratively synthesized and presented in tables and figures. The knowledge gained from this review is expected to provide systematic evidence supporting AI-based predictive models that combine wearable devices for AD, potentially offering insights into model construction details such as data collection and external validation. **(Lines 26-45)**

**g.** Once again, we sincerely appreciate the constructive and valuable feedback provided by the reviewer. We wish you continued success in your work and good health.

------------------------------------------------------------------------------------------------------
2. The paper explores a relatively emerging intersection of wearable devices and AI in Alzheimer's disease (AD). However, the existing literature on AI in healthcare, including Alzheimer's, has been growing, and the manuscript does not highlight its unique contribution clearly in the introduction. It would benefit from a clearer statement on how the review will fill existing gaps.

**Response to Reviewer #1's comment 2:**

Thank you once again for your insightful and constructive feedback. In the previous round of revisions, we addressed how this study will fill the existing gaps in the literature within the introduction section. In this round, we have made further detailed revisions to the introduction, specifically re-writing how our protocol aims to bridge the research gaps. This has been divided into two sections: identifying the gaps in existing reviews and outlining how our protocol will address these gaps.

a. First, it is important to note that this study is a protocol, not a complete review. A protocol outlines the plan for conducting a review, detailing the specific strategies and hypotheses that will be implemented.
b. Therefore, the research gaps addressed by this study are based on our research experience and the potential contributions expected from our planned framework.
c. Through extensive review of the literature, we have identified that existing reviews on the application of AI and wearable devices in Alzheimer's Disease (AD) are incomplete. Specifically, there is a lack of detailed discussion on data collection processes, AI model construction, and feasibility analysis of integrating wearable devices with AI technologies. Our framework addresses key details such as data collection, handling missing data, sample balancing strategies, and model comparisons, with a strong emphasis on the necessity of external validation. These subtopics, such as data collection, involve nuances such as variations between different devices and tasks (even within the same task, such as single-task vs. dual-task gait analysis, where significant differences exist). AI model construction includes areas like feature types, feature selection, algorithm choice, and model comparisons. These and other topics will contribute to the comprehensive content of our forthcoming review.
d. Similarly, this protocol serves as a framework or preliminary version for the future scoping review, similar to the design of a research study. It can also be seen as a placeholder for the topic, preventing redundancy in research and minimizing unhealthy competition, akin to the role of preprints on platforms like arXiv.
e. The research gap we are addressing is providing a detailed workflow from data collection to external validation of models. This will offer a comprehensive overview of the current state of model construction. This approach will help evaluate the mainstream methods, identify key improvements made thus far, and provide a clear understanding of existing research. Specific topics for analysis and discussion include differences in wearable device types and data collection protocols, key digital biomarkers for various types of data, whether missing data handling and sample balancing are common in current research, and performance differences among traditional algorithms, deep learning models, and complex ensemble models. Additionally, we will delve deeper into the status of external validation in existing research.

**f.** Furthermore, each AI model constructed in the studies reviewed will be assessed using the PROBAST tool to evaluate their risk of bias and applicability. This is an important aspect that has not been thoroughly addressed in previous research.

We believe that these detailed revisions will further enhance the clarity and value of our protocol and hope they align with your expectations.

**g.Modified content:(Lines 117-149)**

| **Existing Gaps in Review Studies** | The potential of these technologies is currently being explored. However, many reviews tend to focus on AI models for traditional biomarkers in AD, failing to address the developmental potential of wearable devices in Alzheimer's. Only two systematic reviews [19, 20] have integrated wearable devices with AI models in the context of AD. Furthermore, these reviews do not adequately demonstrate the feasibility of integrating wearable devices with AI technologies. In their descriptions of wearable devices, they fail to provide detailed information on data collection processes, such as device selection, specific collection strategies and paradigms, as well as the dropout and data loss rates during the collection of different types of data (e.g., gait, eye movement, neurophysiological data), and the reasons for these issues (e.g., device malfunction, participant dropout, or non-standard data collection).  Regarding AI models, these reviews lack specific details on model construction, such as feature selection strategies, handling of missing data, sample balancing strategies, comparisons of different algorithm performances, and validation methods. These details are crucial for building wearable device-based machine learning models in Alzheimer's disease. |
| --- | --- |
| **Gaps Our Protocol Aims to Address** | To fill these gaps, a comprehensive review of existing literature is needed. This review should provide a detailed analysis of the workflow from data collection to external validation of models, offering a comprehensive overview of existing model construction. It will help to understand the current state of research, analyze mainstream methods, and evaluate key improvements made so far. Specific topics for analysis and discussion include the differences in wearable device types and data collection protocols, key digital biomarkers for different types of data, whether missing data handling and sample balancing are commonly performed in current research, and the performance differences between traditional algorithms, deep learning models, and complex ensemble models. Additionally, the status of external validation in existing research should be examined in more detail. The AI models constructed in each study will be evaluated using PROBAST to assess their risk of bias and applicability.  These findings will provide valuable insights for future work, including whether systematic reviews and meta-analyses should be conducted, the development of new models, or the validation of the applicability of existing models. Given the breadth of this goal, a scoping review is the most appropriate approach [21]. |

**h.** Once again, we sincerely appreciate the constructive and valuable feedback provided by the reviewer. We wish you continued success in your work and good health.

------------------------------------------------------------------------------------------------------

3. The protocol promises to synthesize a significant amount of existing research, but it remains unclear what specific contributions it will make beyond summarizing previous findings. It would be useful to further emphasize how the findings will directly influence future research directions or clinical practices. Some workss are worth mentioning: (1)DOI:10.62762/CJIF.2024.876830 (2)DOI:10.62762/TIS.2024.680959

**Response to Reviewer #1's comment 3:**

**a.** We greatly appreciate your thoughtful suggestions. In the most recent revision, we have further emphasized how our research findings can directly influence future research directions and clinical practices. We believe this approach will enhance the practical implications of our work.

**b.** Additionally, this study is a protocol, which outlines the steps, methodologies, and presentation of research results. The contribution of this protocol will only be fully realized upon its implementation, with the outcomes expected to be substantiated through future research. While the current contribution is based on hypothetical scenarios, these assumptions are grounded in a thorough review of existing literature and comparisons with similar studies that have overlooked certain aspects. Our research design aims to provide insights that differ from those of prior work.

**c.** As you rightly pointed out, our protocol does summarize existing studies. However, isn't the essence of a review to synthesize the current research and distill novel insights and perspectives from it?

**d.** Comprehensive Analysis of AI and Wearable Devices in Alzheimer’s Disease Prediction: This protocol will systematically evaluate the integration of AI algorithms with wearable devices in Alzheimer’s Disease (AD) prediction models, filling a gap in the existing literature on the combination of these technologies and methodologies.

**e.** Refining Wearable Device Selection and Data Collection Methods: The study will analyze the selection of different wearable devices and the data collection process in detail, including device selection criteria, data collection strategies, and handling of data loss and missing data. This analysis will provide valuable guidance for future device selection in AD prediction and help identify challenges in data collection.

**f.** Transparency in Model Construction and Validation: The protocol will delve into the construction of AI prediction models, covering feature selection, algorithm choice, model training, and external validation. This transparency will assist both researchers and clinicians in understanding how to build and optimize AI-based prediction models, enhancing their applicability in clinical practice.

**g.** Multimodal Data Integration in Application Research: The protocol will also explore the integration of multimodal data (such as physiological signals, gait, and speech data) to investigate methods for combining different data sources. This will enhance model diagnostic accuracy and contribute to more effective predictive modeling.

**h.** Addressing Limitations of Traditional Biomarker Models: While current reviews primarily focus on traditional biomarkers, our protocol will address this gap by providing a comprehensive review of AI and wearable device-integrated prediction models, advancing this emerging field.

**i.** Advancing Early Diagnosis and Personalized Medicine in AD: By offering systematic evidence, this protocol has the potential to provide new perspectives on the early diagnosis and disease progression prediction of Alzheimer’s disease, thereby supporting the development of personalized medical strategies.

**Modified content:(Lines 458-512)**

| **Comprehensive Analysis of AI and Wearable Devices in Alzheimer’s Disease Prediction** | In summary, the contribution of this protocol lies not only in summarizing existing research but also in advancing the application of AI and wearable devices in Alzheimer’s disease (AD) diagnosis and management. This work fills gaps in current research and provides guidance for future studies and clinical practice. Firstly, the planned review study comparing the performance of different algorithms in AD diagnosis will offer clear directions for future research. By identifying which algorithms are more effective for specific tasks, researchers can focus on optimizing these algorithms, thereby improving diagnostic accuracy and predictive capabilities. For models facing performance bottlenecks, further research could concentrate on improving model architectures or adopting new algorithmic strategies. This would not only enhance algorithm efficiency but also potentially expand their application in diagnosing other related diseases. For instance, to address the limitations of deep learning algorithms in data-scarce scenarios, future studies could explore the use of transfer learning or data augmentation techniques to improve performance on small sample datasets. |
| --- | --- |
| **Refining Wearable Device Selection and Data Collection Methods** | Secondly, this protocol emphasizes research on pairing wearable devices with clinical tasks, highlighting the strong correlation between devices and tasks. Future studies, based on the summary provided by the protocol, can further explore which types of tasks are best suited for pairing with specific devices, thus increasing the efficiency of clinical applications. This finding will provide valuable guidance to device developers, helping them create more precise and efficient diagnostic tools. Additionally, issues with missing data during the data collection process offer direction for improving data acquisition technologies. Future studies could explore new data collection methods and technologies, such as the integration of augmented reality (AR) or virtual reality (VR) with wearable devices, to capture richer physiological and behavioral data, thereby providing more data for early AD diagnosis. |
| **Impact of Diverse Data Collection Methods on Model Performance** | Furthermore, by comparing models based on different collection tasks, devices, modalities, and data types, we can gain a deeper understanding of how diverse data collection methods influence model performance. Different collection tasks and devices directly impact data quality. Through the study of these diverse data collection methods, we can uncover how various factors interact synergistically, enhancing the model's performance across different application scenarios. This is particularly relevant in the fields of healthcare and health management, providing both theoretical and practical support for optimizing existing models and developing accurate diagnostic tools. |
| **Application of Multimodal Data Integration in Research** | Moreover, regarding multimodal data integration, this research will emphasize the impact of application differences and performance distributions across diverse datasets, encouraging future studies to focus on effectively combining multi-source data. For example, future studies could explore how to deeply fuse different types of data (such as behavioral data, electroencephalogram (EEG) data, and physiological signals) to further enhance diagnostic accuracy. In clinical applications, these findings will help clinicians understand the relationship between different devices, algorithms, and tasks, thereby enabling more personalized treatment plans in clinical practice. |
| **External Validation and Evaluation of Model Generalizability** | Additionally, regarding external validation and model generalization, the review based on this protocol will highlight future research focus areas, particularly how to improve models’ adaptability across different clinical environments, especially with respect to cross-hospital and cross-regional dataset generalization. Research in this area will foster collaborative studies across institutions, promote the sharing and application of medical data across regions, and provide a foundation for the standardization and precision of AD diagnosis globally. |
| **The Importance of Code Sharing for Bridging the Gap Between Academia and Clinical Practice** | Finally, the initiative of publicly releasing the code will serve as a bridge between the academic and clinical communities. By increasing transparency and reproducibility, researchers and clinicians will be able to more easily validate results, optimize algorithms, and customize development based on varying clinical needs. This will facilitate the deeper application of AI in healthcare, ultimately improving the diagnostic efficiency and accuracy of diseases such as AD. |
|  | Overall, conducting the further review according to this protocol not only provides strong theoretical support for technological advancements in AD diagnosis but also offers feasible pathways for optimizing clinical applications and integrating multimodal data. These findings will directly drive future research directions and help clinical practice achieve greater breakthroughs in early disease detection and personalized treatment. |

**j.** The research you mentioned has already been incorporated into the latest revision of our manuscript.

**Modified content:** Similarly, graph neural networks play a crucial role in the spatiotemporal processing of EEG data by simultaneously considering the dynamic changes in the time series and the spatial relationships between different brain regions[17,18].

**(Lines 104-107)**

------------------------------------------------------------------------------------------------------

4. The manuscript discusses the types of data (e.g., gait, EEG) that will be covered, but there is limited discussion on how diverse data collection methods could influence the model's performance. It should address how the variety of data sources will be handled, especially multimodal data.

**Response to Reviewer #1's comment 4:**

Thank you for your detailed suggestions. In fact, in the previous revision, we already addressed how to handle various data sources. However, we acknowledge that our explanation was overly simplified, and we have now expanded this section with more specific details. We hope that our revisions meet your expectations.

a. First, we have further elaborated on how to handle various data sources and how to categorize and compare the performance of diverse data collection methods. We have classified and compared model performance based on modalities, data types, unified data types from different devices, and tasks. This approach aims to determine the performance distribution of diverse data collection methods.

**Modified content:(Lines 407-428)**

Regarding the performance of models built with different types of data, we will first present the performance distribution differences between unimodal and multimodal models, covering metrics such as accuracy, specificity, sensitivity, and AUC. For studies where performance data is not reported, only the publicly available performance metrics will be compared. In each study, we will compare the performance differences between the models with the best reported performance. In multimodal studies, unimodal and multimodal models are typically constructed and compared, with specific performance metrics provided to highlight the differences in performance between these models. The best data type combinations will be summarized.

Additionally, we will conduct a comparative analysis of studies based on different data types, such as comparing the performance differences between multimodal and unimodal gait models. These differences will provide further insights into each type of model.

Furthermore, regarding the impact of data collection tasks and devices on performance, we will compile the combinations of collection tasks and devices based on the classification of each model type. This will allow us to compare which devices provide the best performance for tasks such as simple gait tasks, under a consistent collection task. We will also examine the performance differences for similar wearable devices across different gait tasks (e.g., single-task versus dual-task gait), thus gaining insights into how diverse data collection methods influence model performance.

**b.** Additionally, we have expanded the discussion section to include the impact of diverse data collection methods on model performance, as well as the influence of multimodal data integration in application research **(Lines 484-500)**.

| **Impact of Diverse Data Collection Methods on Model Performance** | Furthermore, by comparing models based on different collection tasks, devices, modalities, and data types, we can gain a deeper understanding of how diverse data collection methods influence model performance. Different collection tasks and devices directly impact data quality. Through the study of these diverse data collection methods, we can uncover how various factors interact synergistically, enhancing the model's performance across different application scenarios. This is particularly relevant in the fields of healthcare and health management, providing both theoretical and practical support for optimizing existing models and developing accurate diagnostic tools. |
| --- | --- |
| **Application of Multimodal Data Integration in Research** | Moreover, regarding multimodal data integration, this research will emphasize the impact of application differences and performance distributions across diverse datasets, encouraging future studies to focus on effectively combining multi-source data. For example, future studies could explore how to deeply fuse different types of data (such as behavioral data, electroencephalogram (EEG) data, and physiological signals) to further enhance diagnostic accuracy. In clinical applications, these findings will help clinicians understand the relationship between different devices, algorithms, and tasks, thereby enabling more personalized treatment plans in clinical practice. |

------------------------------------------------------------------------------------------------------

5. What are the newly-added values?

**Response to Reviewer #1's comment 5:**

A. First, this study is a protocol that provides a framework for the forthcoming standardized review. It aims to guide the next steps in ensuring a systematic and comprehensive evaluation.
B. Additionally, the protocol outlines our future plans to utilize PROBAST for model assessment, allowing us to assess the risk of bias and applicability of the models. This approach has not been addressed in previous similar reviews.
C. Furthermore, our protocol is expected to generate a series of new insights. As mentioned in your second and third points, the fundamental contribution of this work is to provide a thorough review of the existing literature, offering comprehensive content on these models, which is currently lacking in other reviews. Our protocol also presents a high level of granularity, offering detailed evaluations of the model development process for wearable AI integrations. We anticipate that the systematic analysis included in the review will emphasize structured assessments of AI models, focusing on device selection, data collection paradigms, feature selection, and model validation processes. In fact, even the more basic analyses—such as reviewing the publication status of research codes and adherence to the Tripod protocol—can provide recommendations to enhance reproducibility in future studies.

**Modified content: (Lines 458-512)**

| **Comprehensive Analysis of AI and Wearable Devices in Alzheimer’s Disease Prediction** | In summary, the contribution of this protocol lies not only in summarizing existing research but also in advancing the application of AI and wearable devices in Alzheimer’s disease (AD) diagnosis and management. This work fills gaps in current research and provides guidance for future studies and clinical practice. Firstly, the planned review study comparing the performance of different algorithms in AD diagnosis will offer clear directions for future research. By identifying which algorithms are more effective for specific tasks, researchers can focus on optimizing these algorithms, thereby improving diagnostic accuracy and predictive capabilities. For models facing performance bottlenecks, further research could concentrate on improving model architectures or adopting new algorithmic strategies. This would not only enhance algorithm efficiency but also potentially expand their application in diagnosing other related diseases. For instance, to address the limitations of deep learning algorithms in data-scarce scenarios, future studies could explore the use of transfer learning or data augmentation techniques to improve performance on small sample datasets. |
| --- | --- |
| **Refining Wearable Device Selection and Data Collection Methods** | Secondly, this protocol emphasizes research on pairing wearable devices with clinical tasks, highlighting the strong correlation between devices and tasks. Future studies, based on the summary provided by the protocol, can further explore which types of tasks are best suited for pairing with specific devices, thus increasing the efficiency of clinical applications. This finding will provide valuable guidance to device developers, helping them create more precise and efficient diagnostic tools. Additionally, issues with missing data during the data collection process offer direction for improving data acquisition technologies. Future studies could explore new data collection methods and technologies, such as the integration of augmented reality (AR) or virtual reality (VR) with wearable devices, to capture richer physiological and behavioral data, thereby providing more data for early AD diagnosis. |
| **Impact of Diverse Data Collection Methods on Model Performance** | Furthermore, by comparing models based on different collection tasks, devices, modalities, and data types, we can gain a deeper understanding of how diverse data collection methods influence model performance. Different collection tasks and devices directly impact data quality. Through the study of these diverse data collection methods, we can uncover how various factors interact synergistically, enhancing the model's performance across different application scenarios. This is particularly relevant in the fields of healthcare and health management, providing both theoretical and practical support for optimizing existing models and developing accurate diagnostic tools. |
| **Application of Multimodal Data Integration in Research** | Moreover, regarding multimodal data integration, this research will emphasize the impact of application differences and performance distributions across diverse datasets, encouraging future studies to focus on effectively combining multi-source data. For example, future studies could explore how to deeply fuse different types of data (such as behavioral data, electroencephalogram (EEG) data, and physiological signals) to further enhance diagnostic accuracy. In clinical applications, these findings will help clinicians understand the relationship between different devices, algorithms, and tasks, thereby enabling more personalized treatment plans in clinical practice. |
| **External Validation and Evaluation of Model Generalizability** | Additionally, regarding external validation and model generalization, the review based on this protocol will highlight future research focus areas, particularly how to improve models’ adaptability across different clinical environments, especially with respect to cross-hospital and cross-regional dataset generalization. Research in this area will foster collaborative studies across institutions, promote the sharing and application of medical data across regions, and provide a foundation for the standardization and precision of AD diagnosis globally. |
| **The Importance of Code Sharing for Bridging the Gap Between Academia and Clinical Practice** | Finally, the initiative of publicly releasing the code will serve as a bridge between the academic and clinical communities. By increasing transparency and reproducibility, researchers and clinicians will be able to more easily validate results, optimize algorithms, and customize development based on varying clinical needs. This will facilitate the deeper application of AI in healthcare, ultimately improving the diagnostic efficiency and accuracy of diseases such as AD. |
|  | Overall, conducting the further review according to this protocol not only provides strong theoretical support for technological advancements in AD diagnosis but also offers feasible pathways for optimizing clinical applications and integrating multimodal data. These findings will directly drive future research directions and help clinical practice achieve greater breakthroughs in early disease detection and personalized treatment. |

------------------------------------------------------------------------------------------------------

6. The manuscript lacks a detailed discussion on the theoretical models that underpin the use of AI in AD prediction. A more comprehensive review of existing theories or frameworks (such as machine learning principles and their adaptation for healthcare applications) would strengthen the theoretical grounding.

**Response to Reviewer #1's comment 6:**

A. Thank you for your detailed suggestions. We have already made revisions in the previous round. In this round of revisions, we have further reviewed the theoretical framework of machine learning in Alzheimer’s Disease (AD) and included additional content on the application of machine learning in healthcare, specifically focusing on wearable models for AD. We have also elaborated on model optimization and selection principles, the challenges of machine learning in AD prediction, application frameworks, and optimization methods. We believe that these additions significantly enrich the theoretical foundation of the manuscript.

**Modified content: (Lines 69-116)**

| **Review of the Theoretical Framework** | The fundamental principles of machine learning can be divided into three major categories: supervised learning, unsupervised learning, and reinforcement learning[10]. In AD prediction, supervised learning methods are particularly widely used due to their ability to train models based on labeled data, enabling the differentiation of various disease states. For example, algorithms such as Support Vector Machines and Random Forests have achieved significant results in tasks such as gait analysis and EEG signal analysis. Unsupervised learning also plays a crucial role in the absence of labeled data, particularly in exploring differences and characteristics of AD subtypes. Clustering algorithms can help researchers uncover potential disease patterns. Reinforcement learning, on the other hand, shows promise in personalized treatment and real-time prediction by optimizing decision-making processes, offering new directions for patient health management. |
| --- | --- |
| **Application of Machine Learning in Healthcare (Specifically Wearable Models for Alzheimer’s Disease)** | In the prediction of AD, wearable devices can provide various types of data (such as physiological signals, gait, and activity levels), and when integrated with machine learning, they enable high-frequency monitoring and personalized diagnosis [10]. Clinical prediction models use multiple variables to estimate current diagnostic outcomes (diagnostic models) or predict future disease outcomes (prognostic models). Several studies have developed predictive models for AD based on wearable devices. For example, Younghoon Jeon used SHIMMER wearable devices to monitor gait data and combined algorithms such as random forests and AdaBoost, achieving an accuracy rate of 72% [11]. Ahsan Shahzad developed a support vector machine model, achieving an accuracy rate of 70% [12]. Kyeonggu Lee utilized wearable EEG signals to build a multilayer perceptron model, achieving an accuracy rate of 74% [13]. In our recent study, we integrated multiple wearable sensors within a three-stage federated learning framework to develop a home-based AD detection system [14]. Additionally, we constructed the EmoMarker system using wearable microphones and depth cameras, achieving a classification accuracy of 81.13% by leveraging motion and acoustic data [15] |
| **Model Optimization and Selection Principles** | Typically, choosing an appropriate machine learning algorithm (such as deep learning, random forests, or support vector machines) and training it based on the characteristics of patient data and clinical needs can optimize the model’s predictive performance. For example, when the model needs to handle complex time-series data (such as gait studies in neurodegenerative diseases), methods like recurrent neural networks and long short-term memory networks have demonstrated superior performance[16]. Similarly, graph neural networks play a crucial role in the spatiotemporal processing of EEG data by simultaneously considering the dynamic changes in the time series and the spatial relationships between different brain regions[17, 18]. |
| **Challenges, Application Framework, and Optimization Methods of Machine Learning in AD Prediction** | Additionally, despite the initial progress in applying machine learning techniques to AD prediction, several challenges remain. First, early symptoms of AD are often difficult to detect, and physiological changes are typically slow, requiring machine learning models to have high sensitivity in identifying subtle changes from low signal-to-noise ratio data. Furthermore, data gaps, individual differences, and the heterogeneity of multi-source data pose significant challenges to model training and validation. Therefore, selecting appropriate features, handling missing data, and optimizing model structures have become key factors in improving prediction accuracy and robustness. |

We would like to express our sincere gratitude for Reviewer 1's thoughtful suggestions. We hope that our revisions meet your expectations.

**Response to Reviewer 2:**
Thank you for recognizing the value of our research and for the significant effort you put into the review process. We wish you all the best in your work.

**Response to Reviewer 3:**
We greatly appreciate your positive feedback regarding the quality of our manuscript. We will diligently follow the protocol to carry out the analysis as outlined.
